# Sex differences in social focus across the life cycle in humans

**DOI:** 10.1098/rsos.160097

**Published:** 2016-04-06

**Authors:** Kunal Bhattacharya, Asim Ghosh, Daniel Monsivais, Robin I. M. Dunbar, Kimmo Kaski

**Affiliations:** 1 Department of Computer Science, Aalto University School of Science, PO Box 15400, Aalto 00076, Finland; 2 Department of Experimental Psychology, University of Oxford, South Parks Road, Oxford OX1 3UD, UK

**Keywords:** sex differences, social capital, social investment, data science, life history

## Abstract

Age and gender are two important factors that play crucial roles in the way organisms allocate their social effort. In this study, we analyse a large mobile phone dataset to explore the way life history influences human sociality and the way social networks are structured. Our results indicate that these aspects of human behaviour are strongly related to age and gender such that younger individuals have more contacts and, among them, males more than females. However, the rate of decrease in the number of contacts with age differs between males and females, such that there is a reversal in the number of contacts around the late 30s. We suggest that this pattern can be attributed to the difference in reproductive investments that are made by the two sexes. We analyse the inequality in social investment patterns and suggest that the age- and gender-related differences we find reflect the constraints imposed by reproduction in a context where time (a form of social capital) is limited.

## 1. Introduction

Most species, and humans in particular, exhibit striking changes in social style across the life cycle, in most cases as a consequence of a shift in emphasis from development to reproduction. In humans, a greatly extended period of socialization, combined with a virtually unique period of post-reproductive (grandparental) investment, adds significant complexity to this. Although this much is obvious from casual observation, we actually know very little about the relative investment that individuals make as they age, or how this differs between the sexes. The last decade has seen a rapid growth and development in the information and communications technology (ICT), which has increasingly aided humans to connect to each other. Among the different channels that have become accessible, mobile phone communication is perhaps the most prominent as regards the number of users [1]. This is the reason mobile phone call detail records (CDRs) have increasingly been used to study various aspects of human behaviour [2–4]. For example, from these CDRs, one can construct egocentric networks that, in turn, allow one to undertake detailed studies of ego–alter relationships and the patterns of social investment that individuals make in the different members of their social networks [5–8].

Previous studies have shown that the frequencies with which individuals contact their friends and family by phone correlate with their face-to-face interactions [9,7]. Both the age and gender of individuals have been found to be important factors influencing their communication patterns in mobile phone networks: the gender and age preferences of egos for their alters, for example, have been found to correlate with their geographical proximity [10]. Furthermore, the dynamics of activation and deactivation of ties between individuals have been found to be different for the two genders and across age [11]. In general, homophily and heterophily, which are known to be factors shaping human social interactions [12,13], have turned out to be important in mobile phone communications, at least as regards to the gender preferences of an ego. In a previous study, it was found that for younger egos, the most contacted alter is of the opposite sex [14]. Taken together, this suggests that, whatever their limitations might be, mobile phone data provide valid and reliable insights into human social patterns.

In this paper, we focus on and seek insight into the evolutionary dynamics of the social fabric of human life histories based on studying their techno-social communication patterns. To do this, we analyse a large mobile phone dataset and study the structure of the individual level or egocentric networks. In general, we focus on their static structure for different non-overlapping periods, ranging from a month to a full year. In everyday life for both humans [15] and other primates [16,17], time represents a direct measure of relationship quality. Because time is limited and social investment is costly in terms of time [18,19], individuals are forced to choose how to distribute that time across the members of their network [7,11,15]. Here, we use cross-sectional data on the frequency and duration of phone calls to examine how the pattern of social investment varies across the life cycle in the two sexes.

## 2. Methods

We analyse anonymized CDRs from a particular operator in a European country during 2007. The CDRs contain full calling histories for the subscribers of this operator (we term them ‘company users’ and subscribers of other operators ‘non-company users’). There are 6.6 million company users and around 25 million non-company users appearing in the CDRs in the full 1 year period. Out of the total set of company users, there are 3.2 million users for whom both the age and the gender are available., and only a single subscription is registered. In this study, we have focused only on the voice calls and excluded SMS entries from the CDRs. We construct the network by assigning a link between an ego–alter pair if there is at least one call event between them during the observed time period. It should be noted that in this study we have not considered who initiated the call. Therefore, the ego networks, we consider, are undirected. In general, we study calling patterns pertaining to pairs for whom age and gender are known. However, when the demographic information of the alters is not important for the analysis, we include individuals for whom this information is not known.

### 2.1 Additional filtering

In the dataset, there are company users for whom multiple subscriptions are found under the same contract numbers. For such users, it is difficult to determine their real age and gender. We bypass this issue by considering the gender and age to be unknown for such users. The stored age of each company user corresponds to the year when the contract was signed; as the starting year of users' contracts ranged from 1998 to 2007, we updated the age of each user according to the number of years between the beginning of the year when the contract was signed and the first day of 2007. For some users, their contract starting date is unknown, so we add the average age-correction in the population, which was 3 years (rounded from the actual value of 3.2).

## 3. Results

### 3.1 Number of alters with the age of the ego

**Figure 1.**
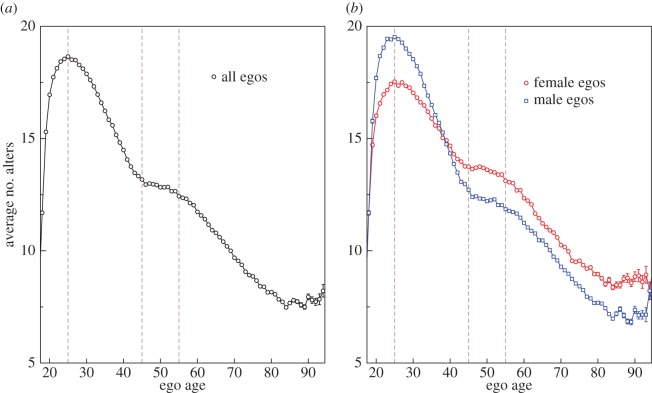
The variation of the average number of alters with the age of the ego (in years). The measurement is done in the time window of a month, and then averaging is carried over the 12 months. (*a*) Egos are considered, irrespective of their sex. (*b*) The behaviour is shown for separate sexes. Male and female egos are denoted by blue squares and red circles, respectively. The error bars span the 95 per cent confidence interval. The dashed lines in the background are used to demarcate different regimes of behaviour (discussed in Results).

**Figure 2.**
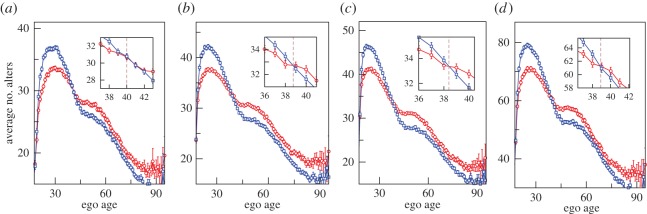
The variation of the average number of alters with the age of the ego for different time windows. The ego networks in (*a*), (*b*) and (*c*) are constructed from CDRs aggregated into the four month periods of January–April, May–August and September–December, respectively. Panel (*d*) corresponds to the time window of the whole year. The figure legend is the same as that in figure 1*b*. The figures in the inset focus on the region where the crossover in behaviour for males and females is found. The dashed lines are used to denote the age of the crossover in each case.

First, we show the variation in the average number of alters that egos contact in a month, as a function of ego's age. From figure 1*a*, we find that the number of alters reaches a maximum at an age of around 25. This is followed by a decrease until an age of around 45. From age 45, the number of alters contacted stabilizes for about a decade. After 55, there is again a steady decrease. In figure 1*b*, we partition these data by gender. From the plot, it is clear that the average number of alters for males is greater than that for females for ages below 39. But from the age of 39 onwards, we observe that the number of alters for females is greater than that for males. To check the robustness of this finding, we use time windows of different length, as shown in figure 2. We observe a consistent pattern and that there is a crossover age at around 39, irrespective of the time window used.

### 3.2 Interaction probability and strength of interaction

**Figure 3.**
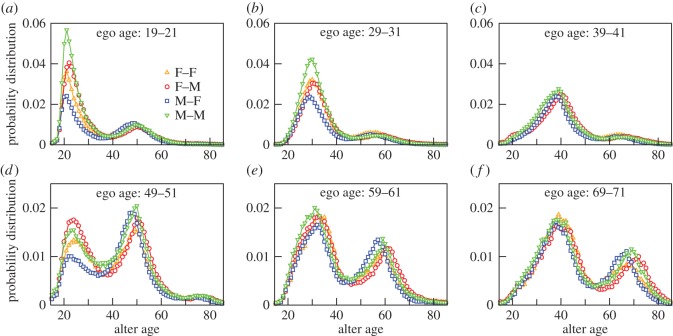
Age distribution of alters for egos and alters of different sexes that participate in phone calls (i.e. the ego networks are undirected). The different plots correspond to the egos' age ranges of 19–21 (*a*), 29–31 (*b*), 39–41 (*c*), 49–51 (*d*), 59–61 (*e*) and 69–71 (*f*) years. In each case, we count the number of alters having different ages and normalize by the total number of alters (male and female). The different symbols and colours used to denote males (M) and females (F) in the ego–alter pairs, are upright orange triangles (F–F), red circles (F–M), blue squares (M–F) and downwards green triangles (M–M). The distributions were calculated over a monthly time window and averaged over 12 months. Only those CDRs were used where the demographic information of the egos as well as the alters was available.

To investigate the interaction pattern of the egos belonging to different age groups, we measure the probability of interaction as a function of the age of alters. For egos of a given age, we find this probability by calculating the number of alters of any age and sex and divide by the total number of alters (male and female). In figure 3, we plot the distribution for egos belonging to the six different age ranges of 19–21, 29–31, 39–41, 49–51, 59–61 and 69–71 years. In general, the distributions appear to be have two peaks. The difference between the ages at which the peaks appear is around 25 years. This is roughly a generation gap and is similar to the results in [14] where the age distribution of the most frequently contacted alter was investigated. Note that the focus of the peaks differs with increasing age: in the younger age groups, the main peak is on individuals of the same age (peers), but from age 50, this starts to be replaced by an increasingly large peak that is a generation younger than ego (presumably ego's now adult offspring). Note how these peaks track each other across the age space as egos' age range changes. Note also the asymmetry in calling pattern between parent and child: the probability of participation of 50 year olds (the parents) with 25 year olds (their adult children) in a call is almost twice as that of 25 year olds with 50 year olds. Interestingly, note also the existence of a third peak with a comparatively small height in figure 3*d* for 50 year old egos. However, the height is only indicative of the fact that the probability for a 50 year old to participate in a call with an alter, one generation older, is much less in comparison than with that of the cohort or their children's age group.

**Figure 4.**
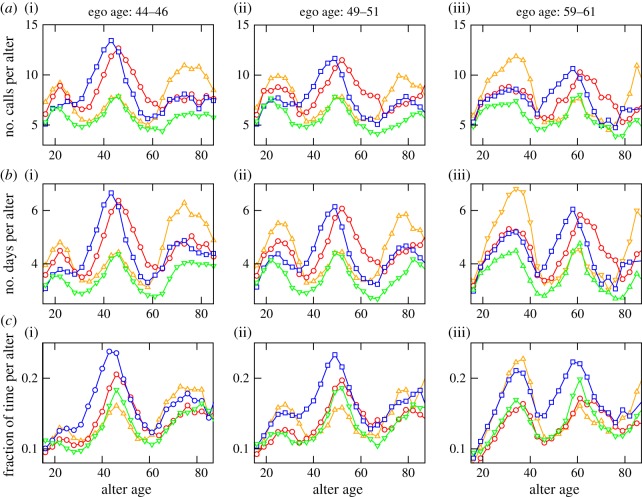
Variation of different quantities characterizing the strength of communication, as a function of the age of alters for the egos' age ranges of 44–46 (*a*,*b*,*c*(i)), 49–51 (*a*,*b*,*c*(ii)) and 59–61 (*a*,*b*,*c*(iii)) years. The different symbols and colours denote the sexes in the ego–alter pairs and are similar to that used in figure 3. The quantities are obtained from monthly call patterns and are averaged over the 12 month period.

To assess the quality of communication with the different alters, we calculate the following quantities: (i) number of calls per alter, (ii) number of distinct days each alter is contacted, and (iii) calling time per alter (time of all calls aggregated within a monthly window). As the total calling time fluctuates strongly, in lieu of (iii), we express the monthly aggregated duration of phone calls to an alter for a given ego as a fraction of the total calling time of that ego. In figure 4, we show these three quantities as a function of the age of the alters, averaged over 12 months for egos' age ranges of 44–46, 49–51 and 59–61. In this computation, the probability shown in figure 3 acts as the normalization (for example, when the total number of calls to a certain age group is divided by the number of alters in that age group we get (i)—shown in figure 4*a*(i), (ii) and (iii)). The plot shows the conspicuous presence of three peaks of comparable heights. For older alters (those aged 70 years or more), the averages are inevitably affected by the small number of older mobile phone users. Nonetheless, in general, the strength of communication appears to be larger when the alter is of the opposite gender and of similar age (compare the plots corresponding with female-ego-to-male-alters (red circles) and male-ego-to-female-alters (blue squares)).

### 3.3 Time budgets of males and females

**Figure 5.**
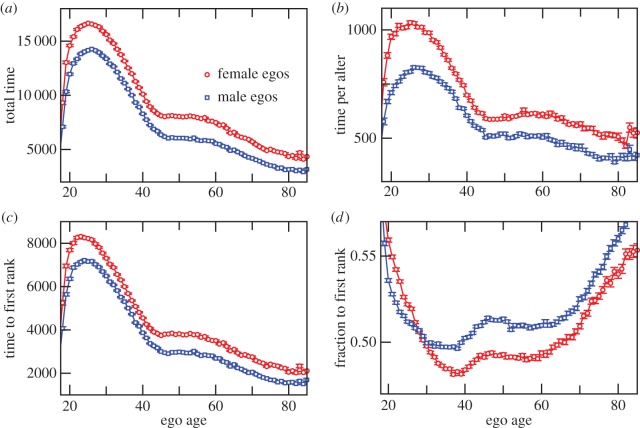
Variation of different quantities characterizing the time budget of egos as a function of their age, for networks constructed in the time window of a month and averaged over the 12 months: (*a*) total time (s) per ego for all calls aggregated in the period, (*b*) time spent (s) per alter per ego, (*c*) time spent (s) per alter per ego with the first-ranked alter and (*d*) the fraction of the total time per ego that is spent with the first rank. Red circles and blue squares indicate female and male egos, respectively.

The structure of the communication pattern of egos is also reflected in the variation in the monthly aggregated call durations. In figure 5*a*,*b,* we plot the total calling time of egos and the calling time per alter, respectively. The plots show that females have larger total calling time as well as larger time per alter than males. Interestingly, the crossover in the number of alters (figure 1*b*) does not get translated to the calling time per alter. For a given ego, we rank the alters in terms of the monthly calling times and plot the calling time to the first-rank alter (figure 5*c*). The time spent by an ego with the first rank is approximately seven times the time spent with an average alter. However, the variation in these quantities is very similar to that of the dependence of number of alters on the age of the ego. When the calling time to the first rank is expressed as a fraction of the total calling time, we observe three broad regimes, a rapid decrease until 40 years of age, a slow variation in the range 40–60 years and a steady rise from 60 years onwards. Note that the variation over the whole age range is only 10% of the average value which is around 0.5. Additionally, a crossover in the behaviour of males and females is visible at the age of 27.

### 3.4 Inequality in call time distribution among alters

Having discussed the dependence of mobile communication upon the gender and age of the egos, we provide a different perspective by measuring the inequality in the way social effort (indexed as calling times) is partitioned among the alters through the Gini coefficient for each ego. The Gini coefficient is mainly used to quantify income inequality and its value varies from 0 (implying perfect equality) to 1 (implying extreme inequality) [20]. We here use it as an evenness index: a Gini value of 0 implies that ego devotes equal amounts of time with all the alters and a value of 1 implies that the ego spends all the time with only one alter.

**Figure 6.**
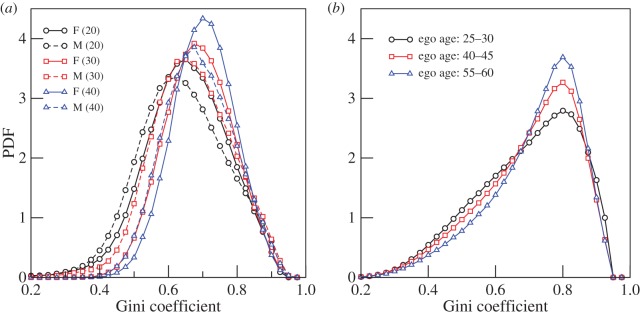
The probability distribution function (PDF) of the Gini coefficient for different sexes and ages of egos. (*a*) Distributions correspond to egos of different sexes (M, male; F, female) and having different numbers of alters (indicated inside brackets). (*b*) Distributions corresponding to different age categories as indicated in the legend. Each distribution is calculated over a month and then averaged over 12 months.

It is natural to expect that there should be a strong bias among the egos with regard to the calling time spent with the alters, during a certain period of time. Here, we analyse the nature of this bias by using the Gini coefficient in two different ways. We note that, for egos of a given age, there is a typical value for the number of alters. This poses a difficulty in comparing two egos of different ages, because the value of the coefficient is known to depend upon the size of the sample set. We circumvent this issue in the following way. First, we consider egos, irrespective of their ages but having a fixed number of alters, and calculate the Gini coefficient for the set of their monthly aggregated call times to the alters. Figure 6*a* shows the distributions for sets of egos having different genders. Comparison between the locations of the peaks suggest that females have overall higher Gini values compared with males.

Next, we consider egos, irrespective of their gender. We choose egos in the age brackets of: (i) 25–30, (ii) 40–45, and (iii) 55–60 years. For each ego, we rank the alters with respect to the time of monthly aggregated call durations. Then, we choose the call times belonging to the top 20 alters. For egos having less than 20 alters, we assume the missing call times to be zero. However, in the analysis, we exclude all egos who have less than six alters. The resulting distribution of Gini values is shown in figure 6*b*. We observe that the inequality among alters is larger for older people than for younger ones. The distributions in figure 6*b* suggest that social effort becomes progressively less evenly distributed as people get older, and that this is true for both genders. In other words, older people devote more attention to their first-ranked alters than younger people do. In effect, younger people are socially more promiscuous, but as they age, they focus more and more of their effort, or social capital, on a smaller subset of meaningful relationships. As it is likely that most of an ego's first few rank alters are family members, this might suggest that older people become more attached to their family compared with younger people. Overall, the female egos exhibit higher inequality values than males do, and this suggests that females may be not only more socially focused than males, but also more attached to their family (as folk wisdom would also suggest).

## 4. Discussion

In order to explore the patterns of social investment across the lifespan in humans, we studied the records of mobile communication belonging to a particular European operator over a 1 year period. As these records include information on the service subscribers' age and gender, we are able to elucidate the nature of the interactions across the life cycle. One important conclusion we can draw is that the average number of contacts is quite modest: in most cases, people focus their (phone-based) social effort each month on around 15 people. This corresponds rather closely to the size of the second layer of egocentric personal networks in the face-to-face world [21,15]. In the face-to-face world, this layer also represents the number of alters contacted at least once a month. Thus, we provide some evidence that the use of mobile phone technology does not change our social world. It also provides further indirect evidence for the fact that we use the phone to contact those who are emotionally closest to us rather than simply those who live furthest away (see also [10]).

Our main finding, however, is the fact that the maximum number of connections for both males and females occurs at the age of around 25 (figure 1). During this early phase, males appear to be more connected than females. After this, the number of alters decreases steadily for both genders, although the decrease is faster for males than for females. The different rates of decrease result in a crossover around the age of 39 such that after 39 females become more connected than males. Note, however, in the age group 45–55, the number of alters stabilizes to a very conspicuous plateau for both males and females. Projecting the slopes for the two graphs before the plateau suggests that the plateau represents a ‘saving’ of around two alters who are retained as monthly alters rather than being lost to the next layer of less frequent contact. The difference between the plateau heights for females and males is around 1.5 alters when the time window corresponds to one month. This difference grows to 3 and 5 alters when the window size is increased to four and 12 months, respectively. Thus, there are two separate but interrelated phenomena: the plateau that appears in both sexes during this period and the difference between males and females in the number of alters contacted. Because this age cohort is that in which ego's children typically marry and begin to reproduce in their turn, one likely explanation for this plateau is that it reflects the fact that parents are maintaining regular interaction with their adult children at a time when some of these might otherwise be lost. The difference between the sexes seems to be primarily owing to the more frequent interactions by the females with their adult children and the children's spouses. Also, females probably interact with their own close family members (e.g. keeping grandparents updated on the children's activities) and the new in-laws created by their children's marital arrangements more than males do.

This shift in women's social focus once offspring reach adulthood and start to reproduce themselves is suggested by the appearance of a rather clear secondary peak in the number of alters aged about a generation (25 years) younger than that appear in the contacts of 50 year olds (figure 3*d*). This is in contrast with the profiles of younger cohorts (those aged 20–40 years) who show a small, but distinct, secondary peak about a generation older than themselves (presumably their own parents). The positions of the peaks in figure 4 tell us quite a lot about domestic arrangements. For example, for this same-age cohort, the peaks in the F–M (circles) and M–F (squares) curves in figure 4 are slightly offset, with the M–F leading by about 3 years. In other words, on average, a woman's main same-age alter is 3 years older than she is, whereas that for a man is about 3 years younger. This is almost exactly the typical age difference between spouses in contemporary Europe, including the country from which our sample derives [22,23]. It seems likely that in figure 4 the peaks to the left are ego's children and the peaks to the right are ego's own parents. This suggestion is reinforced by the fact that these peaks track each other across the age space as ego ages.

In addition, we found another crossover when we looked at the fraction of the total calling time devoted to the top ranked alter. This crossover occurs during the reproductively active period and its location roughly corresponds to the maxima in figure 1. Note, that before the crossover, the fraction for females, in figure 5*d*, is larger than that for males, even though their maximum number of alters is actually lower. As the most frequently contacted alter is typically of the opposite sex [14], we assume this to be the spouse. Because the time costs of reproduction in humans are very high (and may continue to be high for nearly two decades until the children reach marriageable age), we expect that females give priority to their spouses rather than to other kinds of peers (siblings, cousins, friends) during this period when their time (and energy) budgets are under intense pressure. As a consequence, they maintain fewer relationships compared with males of the same age whose investment in their preferred alter seems to be much lower. A similar pattern of withdrawal from casual relationships so as to invest their increasingly limited available time in core relationships as time budgets are squeezed by the foraging demands of parental investment (in this case, lactation) has been noted in baboons [24,25].

These results also seem to reflect female mate choice, with females persistently targeting their spouse in order to maintain investment in their chosen mate once they have made a choice (see also [14]). Note that, when examined over the whole age range, the fraction varies little and remains around 0.5 (figure 5*d*). This observation suggests that across the lifespan, the fractional allocation for the top ranked alter (the spouse) remains conserved even though the absolute time budget decreases (as can be seen from figure 5*a*). This is reminiscent of the finding by [7], who reported, for a much smaller dataset, that the proportional distribution of social effort across all alters in an ego's network remains remarkably constant over time despite considerable change in network membership.

More generally, figure 6 suggests that there was a marked difference in the evenness with which the two genders distributed their social effort, as well as a progressive shift towards being less even with age. Females seemed to be generally more focused in their social arrangements than males, targeting more of their social effort onto fewer alters. This is reminiscent of the finding in [26] that women appear to have a small number of extremely close same-sex friendships, whereas males do not (they typically have a larger number of more casual same-sex friendships). In addition, both genders exhibit the same tendency to shift from being more socially promiscuous (a more even Gini value) early in life to a more uneven (higher Gini value) in their 40s. Because family dominate the inner layers of most people's social networks [15], this would suggest an increasing focus on family and close friendship relationships with age. This might reflect the fact that family relationships are more robust and resilient than friendships, as well as the fact that they are much more important as sources of lifelong support [27,28]. By contrast, the greater social promiscuity of younger individuals could be interpreted as a phase of social sampling in which individuals explore the range of opportunities (both for friendships and for reproductive partners) available to them before finally settling down with those considered optimal or most valuable. In this respect, the younger individuals may be viewed as ‘careful shoppers’ [29] who continue to check out the availability of options, only later concentrating their social effort on a select set of preferred alters.

One implication of this is that turnover (or churn) in network membership might start to fall dramatically at a particular point in the life cycle marked by a shift from this more promiscuous phase to the more stable phase associated with a reduced social network. Figure 1 suggests that the mean number of alters contacted falls from 15–20 during this early phase to 8–15 after age 40. Figures 1 and 5*d* suggest that this switch in social focus may start to occur by the end of the third decade of life, and may thus coincide with the onset of reproduction. The average age of women at first birth in Europe for the currently reproducing generation is around 29 [30] and would fit well with this prediction.

These patterns will, of course, reflect the particular life-history arrangements characteristic of twenty-first century Europe. Where life-history schedules are different, as they are under other socio-economic regimes in the developing world or among hunter–gatherers, we can expect the peaks and patterns in communication to shift to different optima. We have no reason to suppose that mode of communication may exert an especially important influence in this respect, however. People will exploit whatever means of communication is available in order to achieve their particular social goals. Thus, we expect the broad principles revealed in this study to apply more or less universally in all cultures. The differences between cultures will probably lie in the timing of peaks and transitions, rather than in the overall patterns themselves.

## Data accessibility

http://dx.doi.org/10.5061/dryad.j82k4.
